# The Association between Emotional Stress, Sleep, and Awake Bruxism among Dental Students: A Sex Comparison

**DOI:** 10.3390/jcm11010010

**Published:** 2021-12-21

**Authors:** Shifra Levartovsky, Soad Msarwa, Shoshana Reiter, Ilana Eli, Efraim Winocur, Rachel Sarig

**Affiliations:** 1 Department of Oral Rehabilitation, The Maurice and Gabriela Goldschleger School of Dental Medicine, Sackler Faculty of Medicine, Tel Aviv University, Tel Aviv 6997801, Israel; shifralevartov@gmail.com (S.L.); soadm27@gmail.com (S.M.); elilana@tauex.tau.ac.il (I.E.); winocur@tauex.tau.ac.il (E.W.); 2 Department of Oral Pathology and Maxillofacial Imaging Oral Medicine, The Maurice and Gabriela Goldschleger School of Dental Medicine, Sackler Faculty of Medicine, Tel Aviv University, Tel Aviv 6997801, Israel; shoshana.reiter@gmail.com; 3 Department of Oral Biology, The Maurice and Gabriela Goldschleger School of Dental Medicine, Sackler Faculty of Medicine, Tel Aviv University, Tel Aviv 6997801, Israel; 4 Department of Orthodontics, The Maurice and Gabriela Goldschleger School of Dental Medicine, Sackler Faculty of Medicine, Tel Aviv University, Tel Aviv 6997801, Israel; 5 The Dan David Center for Human Evolution and Biohistory Research, Sackler Faculty of Medicine, Tel-Aviv University, Tel Aviv 6997801, Israel

**Keywords:** dental students, bruxism, sex, emotional stress

## Abstract

Psychosocial factors may play an important role in the etiology of sleep and awake bruxism. The purpose of this study was to assess the relationship between emotional stress and bruxism in male and female dental students at various stages of their education. Dental education in Israel is based on a six-year curriculum, divided into three stages: pre-medical studies (yr. 1–2), manual skills (yr. 3–4), and clinical experience (yr. 5–6). Each stage requires different capabilities and skills. Questionnaires regarding psychological state (SCL-90) measuring depression, anxiety, and somatization as well as stress evaluation questioners (Perceived Stress Scale questionnaire 14) were completed by 387 dental students in the 1st to 6th years. Sleep and awake bruxism were evaluated based on the respondent’s awareness. During the manual stage of studies, a significant increase was identified, albeit with weak correlations, between stress scales, depression, anxiety, somatization, and the prevalence of awake bruxism, particularly among males. Only in females was sleep bruxism correlated with emotional parameters, whereas no significant difference in sleep bruxism was observed in males throughout the stages of the study. The manual years of dental education were found to be linked to higher levels of emotional distress and awake bruxism, particularly in men. Sleep bruxism, on the other hand, was not directly linked to emotional factors, implying a distinct etiology.

## 1. Introduction

Stress is concurrently a stimulus and a reaction that includes both physiological and psychological components, which might affect normal functioning [1]. Medical education is perceived as a stressful time as it presents both theoretical and clinical challenges for the students. Stress during education can lead to mental distress and has a negative impact on cognitive functioning and learning. In fact, in recent decades, numerous studies worldwide considered the impact of stress during the period of dental studies. The issue has been studied in dental schools in the US, Europe, India, Saudi Arabia, and Columbia, to name only a few [2]. A systematic review published in 2011 determined that the major sources of dental students’ stress were examinations, clinical requirements, and dental supervisors [3]. This was reinforced by another systematic review and meta-analysis, published in 2014, which demonstrated that stress experienced by dental students is mainly due to the demanding nature of their training [4]. The study also showed how stress during this period could have potentially adverse effects on students’ health and wellbeing, affecting their academic performance, psycho-emotional wellbeing, and physical health. The signs and symptoms of stress may include anxiety and depression as well as somatic symptoms such as upset stomach and sweating [3]. Yet, these symptoms varied between students, with some differences between female and male dental students observed [4]. In addition, differences were identified in the way stress caused by academic, clinic-related, social, and financial factors affected students of different sexes and at different stages of their education [5]. College or university undergraduate students were found to be prone to stressful conditions and, therefore, were more likely to have a high risk of anxiety- and depression-related clinical disorders [6,7,8,9,10,11].

The side effect of stress may be sleep bruxism (SB) and/or awake bruxism (AB), which are masticatory muscle activities that occur during sleep (characterized as rhythmic or non-rhythmic) and/or wakefulness (characterized by repetitive or sustained tooth contact and/or by bracing or thrusting of the mandible), respectively. In otherwise healthy individuals, bruxism should not be considered as a disorder but rather as a behavior that can be a risk and/or protective factor for certain clinical consequences [12].

The prevalence of AB is reported to be 20% among the adult population. The physiology and pathology of AB is unknown, although stress and anxiety have been correlated with its presence and are considered to be risk factors [13]. It probably has a multifactorial etiology, with an interaction of biological and psychosocial factors [14]. Câmara-Souza et al. showed that college preparatory students demonstrated moderate frequency of AB, which was significantly correlated with psychosocial factors [15].

SB can affect approximately 13% of the adult population. Its occurrence is highest in childhood, 14–20%, and decreases with age [16,17,18]. In a review of the literature, Manfredini and Lobbezoo demonstrated that although AB seemed to be associated with psychosocial factors and symptoms, there was no evidence to correlate SB with these factors [19]. In another study, the intensity of sleep bruxism was not found to be significantly correlated with self-reported perceived stress and depression [12,16].

Here, based on the reported differences in stress symptoms between female and male dental students [4], along with the possible association between stress and the development of SB and AB, we aimed to assess whether awake/sleep bruxism is related to sex and emotional stress. For this purpose, we evaluated the associations between stress and subsequent emotional (e.g., anxiety, depression) and somatic (e.g., somatization) consequences and sleep and awake bruxism among male and female undergraduate dental students at various stages of dental education in Israel. The null hypothesis was that the associations between the psychological parameters and sleep and awake bruxism would not be affected by sex difference nor by the different stages of dental education.

## 2. Materials and Methods

The sampled population included 387 students at the Goldschleger School of Dental Medicine, Tel Aviv University. Approval was obtained from Tel-Aviv University Institutional Ethical Committee (No. 100813). Written and oral informed consent was provided by all the participants.

The study consisted of self-report questionnaires delivered to students in the pre-medical, manual, and clinical stages of their dental education. All students were approached in person by an examiner who was not part of the faculty teaching staff at the time (S.M or R.S), and was asked to complete several questionnaires as detailed below. It was made clear that participation was voluntary and, in any case, would be anonymous and have no impact on their academic studies.

Dental education in Israel is based on a 6-year curriculum, divided into 3 stages: the first and second years that include the Pre-medical studies (PreMed) that consist of theoretical courses (e.g., biology, pharmacology, anatomy, etc.); the third and fourth years that include specific sciences related to dentistry (e.g., dental histology, dental morphology, simulation lab courses) with a focus on developing manual skills through practicing on plastic teeth in simulation labs (Manual); and the fifth and sixth years that mainly consist of developing clinical experience by performing various supervised treatments on patients (Clinical).

Group characteristics: 435 students were approached, and 89% of whom responded (*n* = 387). The students’ mean age at the onset of their education was 24 ± 2 years, 180 males and 207 females: 173 students in the PreMed stage (98 males, 75 females), 143 students in the Manual stage (61 males, 82 females), and 71 in the Clinical stage (31 males, 40 females). No significant difference was found between the average age of males and females.

Perceived Stress Scale Questionnaire 14 (PSS14): the level of emotional stress was measured using the PSS, developed by Cohen et al., 1983 [20]. The questionnaire consists of 14 items and examines stressful feelings and thoughts experienced by the respondent over the previous month [14,21,22,23]. The respondent was asked to rate the frequency at which he/she experienced such feelings or thoughts on a scale of four ranging from “never” to “very often,” with a resulting total score ranging between 0 and 56 (a higher score indicated a higher level of emotional stress).

Symptom Checklist-90 (SCL-90) (validated Hebrew version) for Psychological State: the SCL-90 was developed by Derrogatis et al. [24] and designed to reflect the psychological symptom patterns of community, medical, and psychiatric respondents [25]. The validated Hebrew version was used [26]. Each of the items was rated on a 5-point scale of distress (0–4) ranging from “not at all” to “extremely.” The Somatization (Som) scale consists of 12 items, the Depression (Dep) scale of 13 items, and the Anxiety (Anx) scale of 10 items.

Sleep and Awake Bruxism: The measurement of SB and AB was depended solely on the respondent’s knowledge, as assessed through the following questions [23,27]: (1) “In the last 6 months, have you been told, or did you notice yourself, that you grind your teeth or clench your teeth when you are asleep?” (Yes, no, unknown) and (2) “In the last 6 months, are you aware that during wakefulness you are clenching or grinding your teeth?” (Never, used to do it but stopped, at least once a week, at least once a day, more than once a day, most of the day). For statistical purposes, the first two answers (i.e., never, used to do it but stopped) were considered as no-AB, while the rest of the answers were documented as AB.

### Statistical Analyses

The statistical analyses were performed using the IBM SPSS Statistic (v21.0, Armonk, NY, USA) software. A two-way analysis of variance (ANOVA) and a series of t-tests were used to determine significant mean differences and relationships among the variables. The ANOVA was used to assess the main effect of each independent variable and possible interactions between SB, AB, the emotional variables (stress, anxiety, somatization, and depression), period of study (PreMed, Manual, and Clinical), and sex. Spearman correlation was used to evaluate the correlation between the emotional parameters and the prevalence of awake and sleep bruxism. Level of significance was set to *p* ≤ 0.05.

## 3. Results

### 3.1. Psychological Parameters, Period of Study and Sex

Each of the psychological parameters (Stress, Anxiety, Somatization, and Depression) was evaluated according to sex and period of studies (PreMed, Manual, and Clinical).

**Figure 1:**
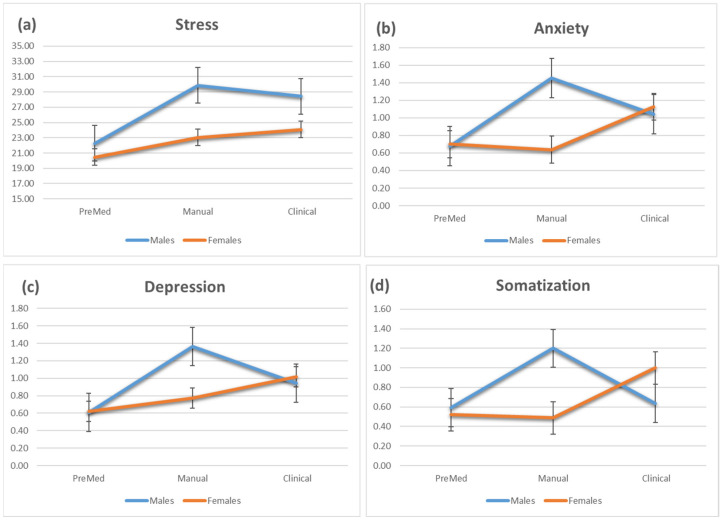
Emotional parameters according to the stage of study and sex: (**a**) Stress, (**b**) Anxiety, (**c**) Depression, and (**d**) Somatization.

Stress: the results showed main effects for period of studies (F _(2,381)_ = 17.379, *p* < 0.001) and sex (F _(1,381)_ = 20.874, *p* < 0.001). A significant interaction was found between period of studies and sex (F _(2,381)_ = 3.621, *p* < 0.03), indicating that males showed a significantly higher increase in stress parameters compared to females in the Manual period (Figure 1a).

Anxiety: the results showed the main effects for period of studies (F _(2,374)_ = 11.02, *p* < 0.001) and sex (F _(1,374)_ = 7.38, *p* < 0.05). A significant interaction was found between period of studies and sex (F _(2,374)_ = 14.06; *p* < 0.001). Males and females started with similar levels of anxiety, but while men’s anxiety increased substantially during the Manual period, women remained unchanged. At the Clinical period, women’s and men’s anxiety was similar (Figure 1b).

Depression: the results showed main effects for period of studies (F _(2,374)_ = 18.57, *p* < 0.001) and sex (F _(1,374)_ = 4.49, *p* < 0.05). A significant interaction was found between period of studies and sex (F _(2,374)_ = 9.01; *p* < 0.001). The pattern was quite similar to that observed for anxiety, with males showing a significantly higher increase compared to females in the Manual period (Figure 1c).

Somatization: the results showed main effects for the period of studies (F _(2,374)_ = 8.22, *p* < 0.001) but no main effect of sex. A significant interaction was found between period of studies and sex (F _(2,374)_ = 16.24; *p* < 0.001). The pattern was quite similar to that observed for former variables, with males showing a significantly higher increase than females at the Manual period (Figure 1d).

### 3.2. AB According to Sex and Stage of Study

**Figure 2:**
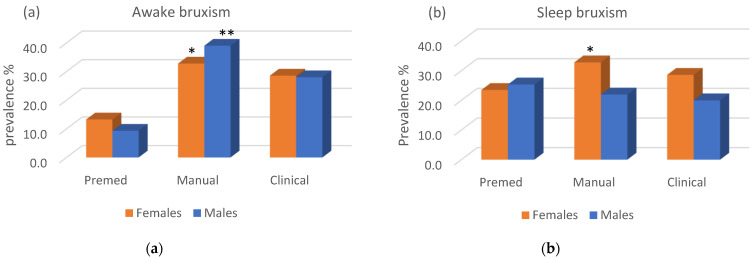
Prevalence of (**a**) AB and (**b**) SB in males and females according to the stage of education (* *p* < 0.05, ** *p* < 0.001).

Overall, out of the entire sample, 23.7% reported having experienced AB. A significantly higher prevalence of AB was noted in the Manual period, as 32.8% of the females (*p* = 0.01) and 39% of the males (*p* < 0.001) reported having experienced AB (Figure 2). Some decrease in AB prevalence was reported for both males and females in the Clinical stage, yet it was still rather high compared to the PreMed stage (9–13%).

Emotional parameters (Somatization, Depression, Anxiety, and Stress) were significantly higher in students reporting AB, mostly in females (Table 1). Males, however, showed a tendency for the same pattern, yet a statistically significant difference was only noted for the Somatization parameter.

Significant, though weak, correlations were found between most of the emotional parameters to the report of AB (Table 2). The highest correlation was found between AB and Anxiety/Depression in females (r = 0.34, *p* < 0.001) (Table 2).

### 3.3. SB According to Sex and Stage of Study

Out of the student’s group, 25% reported SB (20.9% responded unknown). Males and females showed different patterns in SB prevalence; females showed a significant increase at the Manual stage (32.8%) (*p* = 0.024) and some decrease at the Clinical stage (28.6%), which were both higher than those reported by males. Males showed a slight reduction in the prevalence of SB during the Manual and Clinical stages (22% and 20%, respectively) yet with no significant difference (Figure 2).

No significant differences in the emotional parameters (Somatization, Depression, Anxiety, and Stress) were found between students who reported SB and those who did not (Table 1). Significant, though weak, correlations were found between most of the emotional parameters and the report of SB in females, yet no such correlations were found in males (Table 2).

Out of the entire studied sample, 7% (*n* = 28) reported both AB and SB, whereas out of the students reporting AB, 30.4% also reported SB.

## 4. Discussion

The current study aimed to combine sex-related emotional reactions to the dental curriculum structure in Israel in order to determine whether the various phase requirements cause different stress responses depending on sex. Furthermore, awake and sleep bruxism was assessed among undergraduate dental students at various stages of their dental education. Our null hypothesis was rejected as sex differences, and different stages of dental education had an effect on psychological parameters and on bruxism.

In terms of the impact of stress on dental studies, our research found that male and female dental students reacted differently to workload at different stages of the program. The most significant difference in reaction between males and females occurs during the Manual phase, according to our findings. Students appear to begin their studies with comparable levels of stress, anxiety, depression, and somatization, with no obvious sex divergence. During the Manual phase of studies, male students experience a significant increase across all measured emotional parameters, revealing the first instance of sex reaction divergence. It is worth noting that, while male students felt the effects of stress, the parameters measured for female students remained unchanged. These findings contradict other previously conducted studies, which reported that female students showed significantly higher stress scores than males [28,29,30,31,32]. Nonetheless, in the current study, the increase in the measured parameters in male students during the Manual phase (level of anxiety, depression, and somatization) was found to decrease during the clinical phase and align itself with female students (whose parameters gradually increased over time) (Figure 1).

Other findings in the current study were the high incidence of AB (clenching) in the Manual stage, especially in females. In the present study, AB occurred significantly more often than SB, in agreement with the Glaros study [33]. This may be explained by the clear differentiation between AB and SB, as was suggested by the international expert consensus published in 2018 [12]. Despite this conceptual difference, it has been demonstrated that their concomitant presence is frequent and that SB may increase the odds for AB and vice-versa [23]. Although this was not studied in this investigation, 30.4% of the students reporting AB, experienced SB as well. The students in the current study were informed of the meaning of bruxism and the different ways to perform it during wakefulness (clenching, bracing, and thrusting) and during sleep (grinding and clenching). The senior students had studied a full course on temporomandibular disorders (TMD) and bruxism, while the pre-medical students viewed a presentation on bruxism as part of the course “Introduction to Dentistry.” According to Lobbezoo et al. [12], self-reported assessment of sleep or awake bruxism remains the primary tool in bruxism research and clinical practice. This is due to the fact that self-reported bruxism is associated with some psychological conditions, such as stress and anxiety.

Positive, although weak, associations between AB and elevated degrees of stress, anxiety, depression, and somatization were noted. These findings are consistent with previously reported studies [15,17,19,23]. While AB is often claimed to be a response to stress and anxiety, other variables may be associated, such as high and low levels of facial pain and oral habits.

Unlike AB, no significant differences in the emotional parameters (Somatization, Depression, Anxiety, and Stress) were found in the current study between students reporting SB to those who were not, although weak correlations were found between most of the emotional parameters and the presence of SB in females. This correlation between SB and psychological factors is controversial in the literature. Some studies claimed that psychosocial parameters, such as anxiety and stress, are frequently associated with SB [23,34], while others found no evidence for such a relation [19,35]. An explanation for these differences might lie in the fact that AB and SB are difficult to distinguish clinically, especially when the diagnosis is conducted through self-reported questionnaires.

The fact that different stages of the curriculum require students to demonstrate and adopt different skill sets lends credence to the notion that the students’ emotional responses may differ from phase to phase. In essence, the first stage of the curriculum (PreMed) does not require students to develop a new skill set as it is mostly theoretical. As a result, as our study demonstrated, students in the pre-medical stage do not exhibit extreme stress reactions. When students progress to the Manual stage of the curriculum, they must learn new fine motor and precision skills, and failures become more common. Indeed, our research found that both male and female students showed increased stress reactions in all the parameters studied. This reaction is expected since these students’ competence and excellence have never been challenged before, and they struggle when required to perform delicate and precise manual exercises.

During the Manual stage, male students’ reactions are far more extreme/significant than those of their female counterparts, and they are more sensitive to critique and respond with increased emotional and somatic reactions to stress. The reaction of female students is milder, with only a moderate increase in stress and depression and almost no increase in anxiety and somatization. It was suggested that men outperform women in some spatial tasks, whereas women outperform men in fine motor skills assessments [36]. This was attributed to gender roles and the work they are accustomed to performing more regularly, as males have traditionally shown a preference for gross motor performance and women for fine motor performance. However, this is probably not the main reason for the increased stress in males during the Manual stage, as these distinctions have grown less prominent over time due to social and cultural changes in western societies [37]. The role of sex differences in motor activity is controversial as it may reflect existing personality variations [38,39] as well as individual cultural differences [39,40] rather than a sex-related alteration.

In the last stage of studies (Clinical), students are required to implement the skills learned in the Manual phase by treating patients. During this phase, male students displayed a decrease in their stress levels and an associated decrease in anxiety, depression, and somatization measurements, aligning their measurements with those of the female students, whose levels of anxiety, depression, and especially somatization increased steadily throughout their studies period. The elevated stress reaction both in male and female students is probably associated with the stress encountered during social interactions with patients and supervisors. Our results were supported by Dedovic et al. [41], who suggested that women might be more sensitive to social interactions and potential rejections, whereas men may be more threatened when their performance or achievements are challenged. Along with the increased levels of stress, there is a progression of oral parafunctions, bruxism, and eventually TMD signs and symptoms. Taken together, these factors cause a substantial impact on dental students that may interfere with their wellbeing. Therefore, successful implementation of support systems is required to address the results provided herein, and place emphasis on both the curriculum’s phase and the sex of the students.

Although we used a valid and reliable assessment of associated psychosocial impairments, the diagnosis of bruxism was performed via questionnaires without any clinical examination or personal anamnesis, which represents the lower grade of diagnosis (“possible”) of bruxism according to an international group of bruxism experts [12]. In addition, the dental curriculum may vary between different countries and universities; therefore, our findings are only representative of settings that are similar to ours. In light of these limitations, our results should be interpreted cautiously, and other clinical studies should be conducted.

## 5. Conclusions

Significant, though weak, correlations were found between most of the emotional parameters and the report of AB. Higher levels of emotional distress and AB have been linked to the Manual years of dental education, particularly in males. SB, on the other hand, was not directly linked to emotional factors, supporting the idea that it has a different etiology than AB.

## Figures and Tables

**Table 1:** Emotional parameters (Somatization, Depression, Anxiety, and Stress) were identified in male and female students either reporting AB/SB or not (student *t*-test).

		Awake Bruxism	No-Awake Bruxism	*p*-Value *	Sleep Bruxism	No-Sleep Bruxism	*p*-Value *
Females	Somatization	0.88 ± 0.89	0.47± 0.45	<0.001	0.42 ± 0.43	0.47 ± 0.49	NS
	Depression	1.05 ± 0.76	0.62 ± 0.53	<0.001	0.52 ± 0.35	0.61 ± 0.54	NS
	Anxiety	1.02 ± 0.61	0.64 ± 0.64	<0.001	0.55 ± 0.35	0.69 ± 0.69	NS
	Stress	25.33 ± 7.21	20.78 ± 8.57	<0.001	21.9 ± 7.3	20.3 ± 9.07	NS
Males	Somatization	1.07 ± 0.85	0.77 ± 0.73	0.03	0.91 ± 0.79	0.77 ± 0.66	NS
	Depression	1.17 ± 0.81	0.93 ± 0.79	0.08	0.94 ± 0.70	0.97 ± 0.76	NS
	Anxiety	1.28 ± 0.86	1.01 ± 0.89	0.06	1.02 ± 0.77	1.04 ± 0.87	NS
	Stress	28.02 ± 7.58	26.32 ± 8.98	NS	27.4 ± 6.2	26.1 ± 8.9	NS

* *p*-Value greater than 0.05 was marked as non-siginficant (NS).

**Table 2:** The association between the emotional parameters and the report of AB/SB in males and females (Spearman correlation).

		Awake Bruxism				Sleep Bruxism			
		Somatization	Depression	Anxiety	Stress	Somatization	Depression	Anxiety	Stress
Females	Correlation (r)	0.19	0.26	0.34	0.21	0.20	0.27	0.20	0.29
	*p*-value *	0.013	<0.001	<0.001	0.005	0.008	<0.001	0.007	<0.001
Males	Correlation (r)	0.16	0.15	0.15	NS	NS	NS	NS	NS
	*p*-value *	0.020	0.036	0.029	NS	NS	NS	NS	NS

* *p*-Value greater than 0.05 was marked as non-siginficant (NS).

## Data Availability

Data available upon request due to restrictions of privacy. The data presented in this study are available upon request from the corresponding author.
